# Effects of Walking in Bamboo Forest and City Environments on Brainwave Activity in Young Adults

**DOI:** 10.1155/2018/9653857

**Published:** 2018-02-11

**Authors:** Ahmad Hassan, Jiang Tao, Guo Li, Mingyan Jiang, Liu Aii, Jiang Zhihui, Liu Zongfang, Chen Qibing

**Affiliations:** College of Landscape Architecture, Sichuan Agricultural University, Chengdu, Sichuan 611130, China

## Abstract

*Background*. In Japan, “Shinrin-yoku” or forest bathing (spending time in forests) is a major practice used for relaxation. However, its effects on promoting human mental health are still under consideration. The objective of this study was to investigate the physiological and psychological relaxation effects of forest walking on adults. Sixty participants (50% males; 50% females) were trained to walk 15-minute predetermined courses in a bamboo forest and a city area (control). The length of the courses was the same to allow comparison of the effects of both environments. Blood pressure and EEG results were measured to assess the physiological responses and the semantic differential method (SDM) and STAI were used to study the psychological responses. Blood pressure was significantly decreased and variation in brain activity was observed in both environments. The results of the two questionnaires indicated that walking in the bamboo forest improves mood and reduces anxiety. Moreover, the mean meditation and attention scores were significantly increased after walking in a bamboo forest. The results of the physiological and psychological measurements indicate the relaxing effects of walking in a bamboo forest on adults.

## 1. Introduction

Stress is a main public health concern that is associated with psychological health problems, cardiovascular problems, burnout syndrome, and neurological and immunological diseases [1]. In modern urban societies, long-term stress and inadequate recovery from stress are documented as an increasing problem and have long-term effects on health [2, 3]. Additionally, psychosocial issues play vital roles in the development of musculoskeletal pain. Existing healthcare practices, however, are expensive and often emphasize treating stress-related illnesses, instead of stopping them. These findings indicate that stress control is a major factor in maintaining good health and alleviating stress-related disease in urban societies. Researchers are increasingly interested in whether nature may support both the alleviation of illnesses that are facilitated by mental processes, such as stress, and the cure of stress-related diseases, such as depression and fatigue. Many studies from Asia, Europe, and North America have reported that compared with city surroundings, natural surroundings improve human concentration and performance [4–6] as well as mood states [7, 8]. Furthermore, widespread observations suggest that natural surroundings can boost human health [9]. Live contact with plants and trees in urban parks or gardens has been shown to relax and calm people [10–13]. In Japan, “Shinrin-yoku,” or forest bathing, is defined as entering a forest environment. The practice is currently receiving greater attention as a means of relaxation and stress reduction. In “Shinrin-yoku” activities performed in forest environments are used to increase psychophysiological health [14]. Furthermore, walking, the most common physical activity, is becoming increasingly important in the prevention of diseases [15–17]. A recent study demonstrated that forest walking can increase self-rated health issues and tends to minimize psychological stress in healthy individuals [18]. Additionally, positive mental benefits can be observed with forest walking in individuals with major depressive symptoms [19]. We have reported the psychophysiological relaxation effects of “forest bathing” from the perspective of brain activity and emotions. In previous studies, psychological responses have been recorded using the Profile of Mood States (POMS) [20, 21]; however, the semantic differential method (SDM) and State Trait Anxiety Inventory (STAI) have rarely been used. Moreover, psychological studies have indicated that humans have an effective response to forest surroundings that is effective for depression alleviation, psychological relaxation, and stress reduction [7, 22–25]. However, the effects on physiological indices such as brain activity are unclear. Previous studies lack scientific, evidence-based research on the therapeutic effects of bamboo forest walking on brain activity. Therefore, this study aimed to clarify the physiological and psychological relaxation effects of walking in a bamboo forest on adults.

## 2. Materials and Methods

### 2.1. Participants

Sixty Chinese university student volunteers (30 males and 30 females; mean age, 19.6 ± 1.42 years) were selected for this experiment. Posters were placed around campus to recruit participants with the following characteristics: (1) students between 19 and 24 years old, (2) students without physical or mental illness, (3) students not taking any drugs, and (4) students who live and work in Chengdu or nearby. Only participants free of any previously identified physical or mental disorders were selected for this experiment, and those who were in poor physical or mental condition were excluded from this study. Alcoholics and smokers were also excluded at this stage. Smoking, alcohol consumption, and vigorous physical activity were forbidden throughout the study period. Before conducting the experiments, detailed information about the rules and study objective were provided to each participant, and written informed consent was obtained from each subject. After receiving a brief introduction of the experiment, the participants visited and previewed the experiment locations in the city area and the bamboo forest. Similar hotel rooms were provided as living accommodations for each participant, and similar food items were provided during this study to stabilize the background study conditions. The study was performed with the approval of the local Ethics Committee of the College of Landscape Architecture, Sichuan Agricultural University, China.

### 2.2. Study Locations

**Figure 1:**
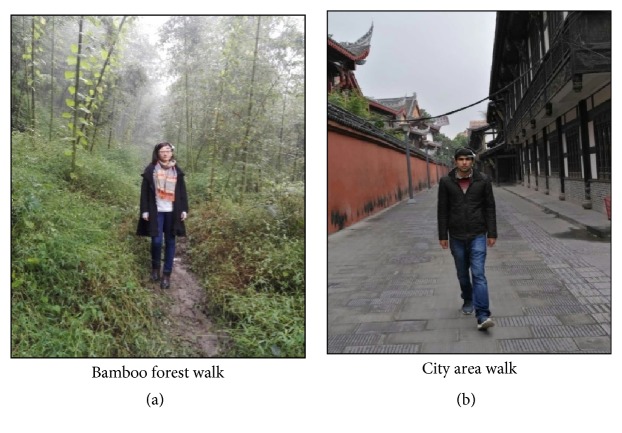
Experimental places.

The experiment was conducted in a bamboo forest near Chengdu and an urban area in Chengdu that included many traditional buildings was selected as the control site (Figure 1). The weather was cloudy on the days that the experiments were conducted. In the bamboo forest and city area, the average temperature and humidity were 22°C, 80%; 27°C, 60%, respectively.

### 2.3. Protocol and Experimental Procedures

All subjects were randomly divided into two groups, that is, groups A and B, consisting of 30 participants each. The subjects moved within their experimental locations once they arrived. Then after resting for 5 minutes, each subject was guided on a walk (15 minutes) in a predetermined area. On the first day of the experiment, 10 participants in the first group walked alone in the bamboo forest for 15 minutes, and the other 10 participants walked alone in the city area for 15 minutes. The time varied slightly depending on the participant. On the second day, each group switched activities. Both activities were performed during the daytime (09:30 AM–11:30 AM). The bamboo forest walking track was arranged in a well-managed bamboo forest area, and the city walking track was located in an urbanized area. All experimental methods were the same for both the city area and bamboo forest locations. All walking tracks were generally flat, except for the slight slope in the bamboo forest, at the beginning, and the course length was the same for both the city and bamboo forest conditions. Each participant's blood pressure was measured 5 minutes before and after each walk (15 minutes) while the participant was seated, whereas the EEG was continuously measured throughout during each walk. The participants completed the STAI and semantic differential questionnaires before and after completing their walk.

### 2.4. Measurements

Blood pressure and EEG results were measured to record the participants' physiological responses. Blood pressure (systolic (mmHg); diastolic (mmHg); and pulse rate) was measured using a sphygmomanometer (Omron, HEM-7011, Omron, China), and EEG results were recorded using a NeuroSky MindWave-EEG headset (Beijing Oriental Creation Technology Co., Ltd., China). The MindWave-EEG headset, which records brainwaves from the Fp1 position (frontal lobe) above the eye [26], is divided into four parts: a headband, an ear clip, a sensor arm containing the EEG electrode, and a Bluetooth device. Two dry sensors are used to filter and detect the EEG signals. The sensor tip identifies electrical signals of the brain from the forehead. The sensor detects ambient noise generated by human muscles, electrical sockets, computers, light bulbs, and other electrical devices. The ear clip acts as a ground and reference, which allows the ThinkGear chip to filter out the electrical noise [27]. The instrument measures the raw signal, power spectrum (high alpha, high beta), mediation level, and attention level. The raw EEG data are detected at a rate of 512 Hz. Other measured values are obtained every second. Therefore, the raw EEG data are the main source of information of EEG signals using the MindWave MW001. The device has small microchips that preprocess data and transfer electrical signals directly to the computer via Bluetooth. The raw EEG data which include high alpha and high beta power units were collected at 1-minute intervals at each experimental site, and 15-minute averages were compared between the two conditions. The headset can detect brainwave signals in the form of meditation and attention scores. According to the EEG e-Sense Metric, attention and meditation data are scaled from 1 to 100 (40 to 60: natural state, 60 to 80: slightly high, 80 to 100: very high, 20 to 40: slightly low, and 0 to 20: very low) [28, 29]. The SDM [30] and STAI [31] were used to study the participant's physiological responses in both environments.

### 2.5. Statistical Analyses

Paired *t*-tests and repeated-measures ANOVA were used to compare the mean values of the physiological data between two sites, whereas the Wilcoxon signed-rank test was used to compare the mean values of the psychological data. The statistical analysis was performed using SPSS 16.0 (SPSS Inc., Chicago, IL, USA) and a *p* value < 0.01 was considered to be statistically significant.

## 3. Results

**Figure 2:**
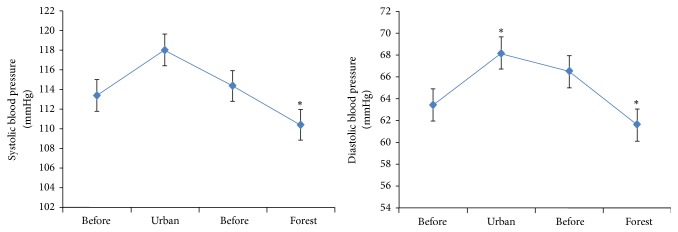
A walking program significantly reduced the blood pressure of subjects. ^*∗*^ *p* < 0.01: bamboo forest walk versus city area walk using a paired *t*-test (*N* = 60).

**Figure 3:**
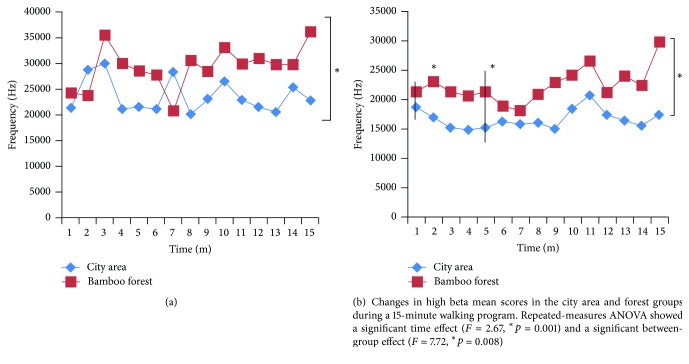


**Figure 4:**
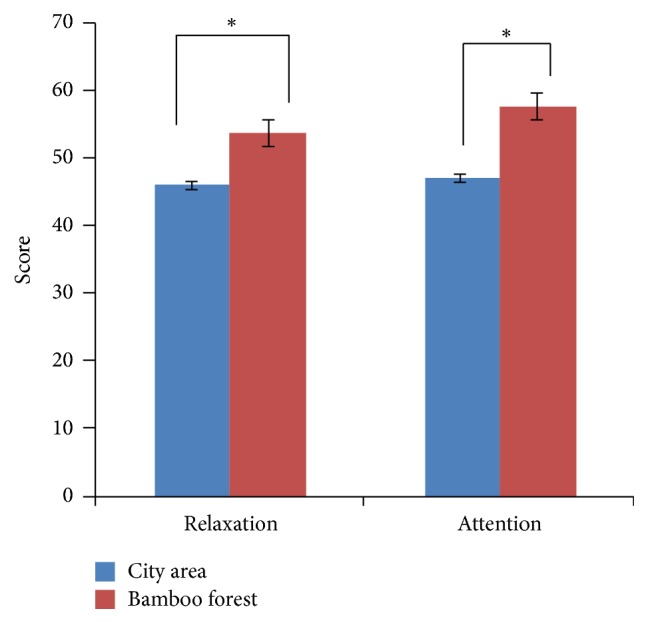
Comparisons of participants' relaxation and attention scores during the bamboo forest and the city area walks. *N* = 60: mean ± standard error. ^*∗*^ *p* < 0.01: determined using a paired *t*-test.

**Figure 5:**
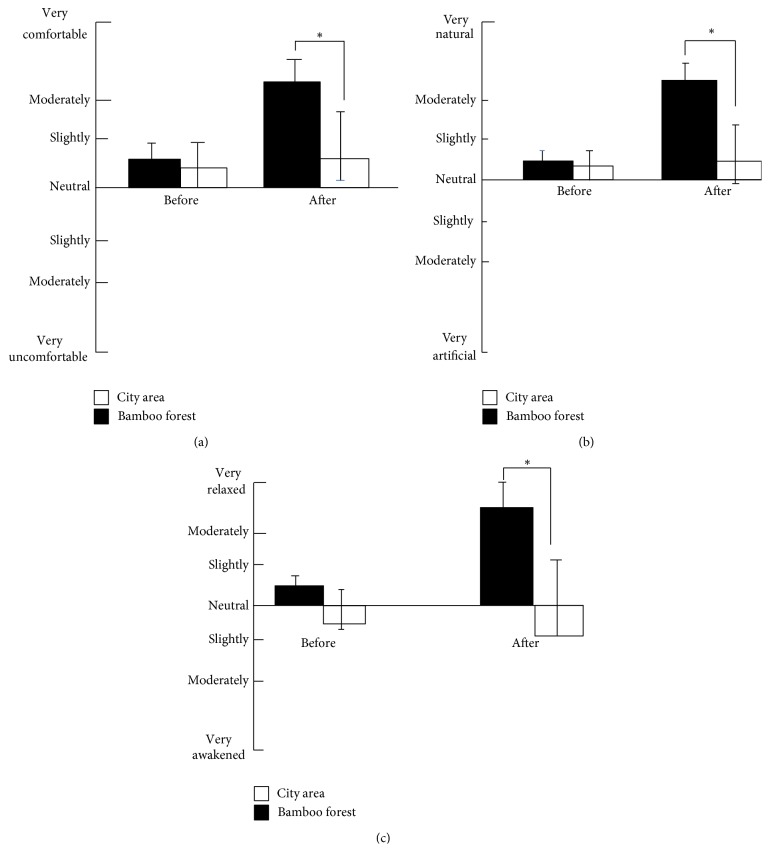
Comparison of subjective scoring for “comfortable” (a), “natural” (b), and “relaxed” (c) feelings before and after walking in the bamboo forest and in the city area. *N* = 60: mean ± SE. ^*∗*^ *p* < 0.01: Wilcoxon signed-rank test.

**Figure 6:**
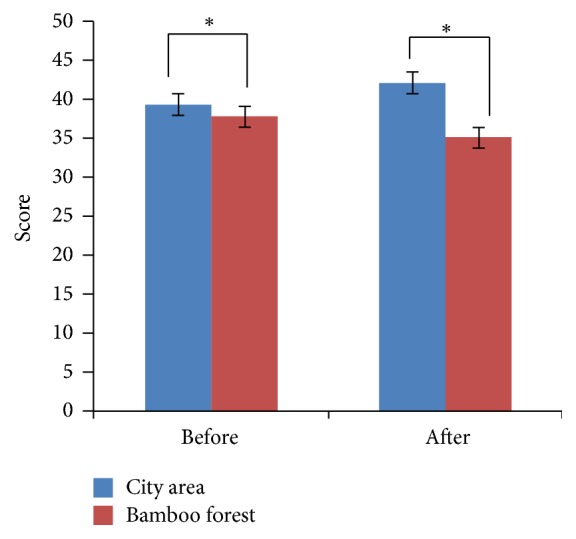
Comparison of the state anxiety scores obtained before and after walking in the city area and bamboo forest environments. *N* = 60: mean ± SE. ^*∗*^ *p* < 0.01: determined using a paired *t*-test.

As shown in Figure 2, participants' systolic (*p* = 0.01) and diastolic (*p* = 0.001) blood pressure were significantly reduced both before and after walking in the bamboo forest. However, the diastolic (*p* < 0.001) blood pressure was significantly higher both before and after walking in the city area (Figure 2). When the results of the EEG high alpha and high beta brainwaves were compared, significant differences were observed under both conditions. During the 1-minute analysis, most of the high alpha brainwave means values were higher when the participants walked in the bamboo forest than when they walked in the city area (Figure 3(a)). A repeated-measures ANOVA comparing the high alpha mean values between the groups with regard to time changes revealed a significant difference (*F*_1,58_ = 12.0, *p* = 0.001), in relaxation between the groups. However, no significant main effect for time was observed within the groups (*F*_14,58_ = 1.53, *p* = 0.09). Moreover, the high alpha mean values changed in the city area (23,585.7 ± 3254.5) and the forest groups (29,249.7 ± 4094.3). Similarly, during the 1-minute analysis, most of the high beta means values were higher when the participants walked in the bamboo forest than when they walked in the city area (Figure 3(b)). According to repeated-measures ANOVA, the beta wave mean values increased in a time-dependent manner (*F*_14,58_ = 2.67, *p* = 0.001), and a significant between-group effect was observed (*F*_1,58_ = 7.72, *p* = 0.008). Using the Bonferroni post hoc test, a difference (*p* < 0.05) was observed between the city area and the bamboo forest groups after 1 and 5 minutes (*p* = 0.05). Moreover, the high beta mean values changed in the city area (16,561.6 ± 1621.4) and the forest groups (22,332.6 ± 2903.8). The subject's meditation and attention mean scores were significantly higher when walking in the bamboo forest than when walking in a city area (meditation score: bamboo forest: 53.6 ± 8.1 and city area: 45.9 ± 10.2; attention score: bamboo forest: 57.5 ± 9.8 and city area: 47.0 ± 8.5; *p* < 0.01, Figure 4). The participant's' SDM and state anxiety scores indicated differences in psychological responses between the two sites. Evaluation using the SDM indicated that the participants felt more “comfortable,” “relaxed,” and “natural” after the bamboo forest walk than after the city area walk both before and after walking. Significantly higher scores were observed for the adjectives “comfortable,” “relaxed,” and “natural” (*p* < 0.01, Figure 5) after walking in the bamboo forest than after walking in the city area. Finally, total state anxiety scores were significantly reduced after bamboo forest walking compared with the city area walking scores (bamboo forest: 35.0 ± 7.39 and city area: 41.9 ± 9.78; *p* < 0.01, Figure 6). However, no significant difference was observed between the city and bamboo forest groups before walking.

## 4. Discussion

In this study, we investigated the relaxation effects of a 15-minute walk in a bamboo forest by examining physiological and psychological changes and comparing the outcomes with those obtained after walking in a city area. Our results indicated that the participant's blood pressure significantly decreased after the 15-minute walk in a bamboo forest, which demonstrated that the forest environment had a significant relaxing effect on the human body. Our results are in some respects similar to those in previous studies examining the relaxing effects of forest bathing on humans [20, 32–34]. Normal physical and habitual walking exercises have a positive effect on lowering blood pressure [35–37]. Park et al. (2010) reported that walking in a forest environment for 20 minutes induced a significant reduction in systolic and diastolic blood pressure compared with the effects of walking in city areas [21]. However, there are many differences in our study; for example, the subjects were different. Our study included young males and females, but only males were included in previous studies. Additionally, the reduction of blood pressure in our study was greater. The physiological measurement results strongly support the findings of previous indoor research using blood pressure and heart rate measurements to evaluate the effect of seeing a forest view on recovery from stress [11, 24]. The findings were also similar to the results of Park et al. (2008), who reported the physiological effects of “forest bathing” using heart rate variability, pulse rate, and salivary cortisol as indicators [38]. Park et al. (2007) reported physiological effects of forest environments using cerebral activity and salivary cortisol as indicators [14]. Tsunetsugu et al. (2007) described physiological effects of forest settings using indicators such as HRV, pulse rate, blood pressure, and salivary cortisol [39]. Additionally, Yamaguchi et al. (2006) described exercise effects of the forest setting based on salivary amylase activity [40]. In this experiment, brain activity was proposed as an indicator of human comfort, and we investigated how high alpha and high beta brainwaves behave in the context of two different environments. The results of the EEG analysis indicated that the participants' overall 15-minute high alpha mean values significantly increased after a walk in the bamboo forest. Thus, according to the increase in high alpha power, we conclude that the forest environment induces mental relaxation. Increased alpha power refers to a state of euphoria and relaxation [41]. In contrast, in the city area walk, lower alpha wave activity was observed; thus, lower alpha wave activity in the city environment indicates that the participants were under stress. An increase in alpha power occurs with feelings of anger and happiness, and a decrease in alpha power occurs with feelings of sadness and fear [42]. During the 15-minute walk, the participants' high alpha waves increased soon after the participants started walking in the bamboo forest. A reduction in high alpha waves was observed when the participants started walking in the city area. We conclude that, with an increase in satisfaction level, the relative power of alpha increases. Alpha band power typically increases when people are in a state of wakefulness and the body is relaxed. Alpha waves are associated with alertness, calmness, learning, and mental coordination [43, 44]. Furthermore, similar conditions are found when recording beta activity. The high beta activity indicated that the participants were more active or highly attentive during their walk in the bamboo forest than during the city area walk. In this case, lower beta wave activity revealed reduced or a lack of attentiveness. Increased beta wave activity is typically associated with an alert condition and decreased beta wave activity is associated with a state of drowsiness [45]. In general, beta waves are fast wave activities that occur during various tasks, such as problem-solving, decision-making, and deep conversation [46]. We conclude that an increase in alpha and beta power is a sign of mental relaxation. An increase in alpha and theta power is observed during meditation and relaxation techniques [47, 48]. The EEG e-Sense Metric indicated that the subjects' meditation and attention scores were higher when walking in the bamboo forest than when walking in the city area. These results indicate that the relaxation and focus levels of participants were higher after a walk in a bamboo forest. These levels are reduced in a city environment because the participants are not fully attentive or might feel bored. Crowley et al. (2010) conducted two psychological computer-based tests to measure attention and meditation levels and found that the MindWave-EEG headset can measure meditation or stress levels over a given time period. The results clearly indicated when the participant undergoes a change in these emotions [49]. According to the psychological questionnaires, the participants in this study felt more relaxed, comfortable, natural, and less anxious after a walk in the bamboo forest. These results are in some respects similar to previous findings describing the effects of a walk in an urban park [20, 33, 50]. Our study results indicated that bamboo forest walking decreased anxiety levels to a greater degree than city walking. One study reported that physical activity decreases anxiety levels [51], indicating that artificial surroundings may reduce the positive health effects of physical activity. The positive effects of bamboo forest walking suggest that it is a simple, attainable, and effective method to improve the quality of life and health of urban dwellers. However, we do not understand the main reason for the different results obtained regarding brain activity. These findings potentially resulted from the differences in environmental conditions, such as the temperature (city: 27°C and bamboo forest: 22°C), humidity (city: 60% and bamboo forest: 80%), or the presence of nature, including plants and the abundance of trees. The benefits of nature play a crucial role in improving mental health; thus, city planners and landscape designers should attempt to maintain green areas to improve the quality of life of urban dwellers. However, the present study has several limitations; for example, only young male and female participants were recruited. Future studies with older adults are required to assess the brainwave mechanism. Additionally, studies on different forest environments are warranted.

## 5. Mechanism of Brainwaves

**Figure 7:**
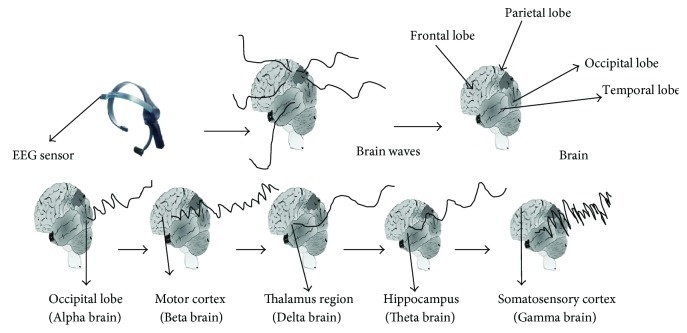
Brainwaves.

Other questions remain, for example, where are brain waves produced? The human brain is divided into the frontal lobe, occipital lobe, parietal lobe, and temporal lobe. The alpha brain wave is a type of wave that can be detected by EEG and originates from the occipital lobe during relaxation. Beta brain waves originate in the motor cortex (frontal lobe). Delta waves are also called high amplitude brain waves and are produced in the thalamus region (reticular formation) or the cortex (suprachiasmatic nuclei). Theta waves are activated during action and originate in the hippocampus. Finally, gamma waves are generated in the somatosensory cortex (the region between the frontal and parietal lobes) (Figure 7).

## 6. Conclusions

Our study results indicated that physical activities in a bamboo forest can have positive effects on brain activity, which supports the belief that forest bathing can be effective for relaxation.

## Additional Points

This project is investigating the coupling mechanism of the human physiological and psychological responses to the ornamental bamboo and its healthy function.
